# Platelets in Skin Autoimmune Diseases

**DOI:** 10.3389/fimmu.2019.01453

**Published:** 2019-07-04

**Authors:** Xiaobo Liu, Christian Gorzelanny, Stefan W. Schneider

**Affiliations:** ^1^Department of Dermatology, Medical Faculty Mannheim, University of Heidelberg, Mannheim, Germany; ^2^Department of Dermatology and Venereology, University Medical Center Hamburg-Eppendorf, Hamburg, Germany

**Keywords:** platelets, autoimmune disease, SLE, SSc, vasculitis, neutrophil, endothelial cell, complement

## Abstract

Systemic lupus erythematosus (SLE), systemic sclerosis (SSc), and small vessel vasculitis are three autoimmune diseases frequently manifested in the skin. They share common pathogenic features, including production of autoantibodies, loss of tolerance to self-antigens, tissue necrosis and fibrosis, vasculopathy and activation of the coagulation system. Platelets occupy a central part within the coagulation cascade and are well-recognized for their hemostatic role. However, recent cumulative evidence implicates their additional and multifaceted immunoregulatory functions. Platelets express immune receptors and they store growth factors, cytokines, and chemokines in their granules enabling a significant contribution to inflammation. A plethora of activating triggers such as damage associated molecular patterns (DAMPs) released from damaged endothelial cells, immune complexes, or complement effector molecules can mediate platelet activation. Activated platelets further foster an inflammatory environment and the crosstalk with the endothelium and leukocytes by the release of immunoactive molecules and microparticles. Further insight into the pathogenic implications of platelet activation will pave the way for new therapeutic strategies targeting autoimmune diseases. In this review, we discuss the inflammatory functions of platelets and their mechanistic contribution to the pathophysiology of SSc, ANCA associated small vessel vasculitis and other autoimmune diseases affecting the skin.

## Introduction

Platelets are small circulating cellular fragments that originate from megakaryocytes mainly within the bone marrow (1, 2). Under physiological conditions, platelets have a short lifespan in the circulation as they are eliminated in the spleen and liver after 7–10 days. Under resting conditions, the vascular endothelium continuously prevents platelet adhesion and activation through the release of prostacyclin I2 and nitric oxide (2). Blood vessel damage or detachment of the endothelium upon injury results in the exposure of the pro-coagulant subendothelial matrix and associated perivascular cells which promote platelet activation and blood clotting. However, platelet adhesion and coagulation could also be initiated without the denudation of the endothelial cell layer. Distinct stimulatory agents such as thrombin, histamine, tumor necrosis factor (TNF-α), or CD40 ligand (CD40L, CD154) convert the endothelium into a proinflammatory and procoagulatory surface through the release of von Willebrand factor (VWF) (3–6). Secreted VWF gets immobilized on the luminal site of endothelial cells where it is activated through blood shear flow mediated stretching. These VWF fibers can rapidly interact with GPIb-IX-V on platelets, resulting in the formation of platelet decorated VWF strings (3, 7, 8). Attached platelets translocate GPIIb/IIIb to their surface to stabilize their interaction with VWF. Moreover, these procoagulant platelets expose phosphatidylserine (PS) on their membrane. Together with tissue factor and Factor VII, PS initiates the activation of the coagulation factors X (FX) and II (FII, prothrombin) (9–13). The presence of tissue factor on platelets is controversial discussed. However, more recent studies suggest its expression and its surface exposure upon activation (14, 15). Apart from tissue factor, platelets can enhance hemostasis through the presentation of P-selectin (CD62P) and lysosomal-associated membrane protein 1 and the release of FV, histamine and ADP (2, 10).

Next to their contribution to hemostasis, there is growing body of evidence indicating the action of platelets in inflammation and immune responses (1, 16–18). Moreover, recent findings point toward the significant involvement of platelets in the pathogenesis of autoimmune diseases (7, 19, 20). This review will describe platelet immune functions, and highlight the implication of platelets in the pathogenic mechanisms of autoimmune disorders with frequent but not limited manifestations in the skin. We will in particular focus on systemic lupus erythematosus (SLE), systemic sclerosis (SSc) and antineutrophil cytoplasmic antibody-associated small vessel vasculitis (AAVs).

## Inflammatory Functions of Platelet

**Figure 1:**
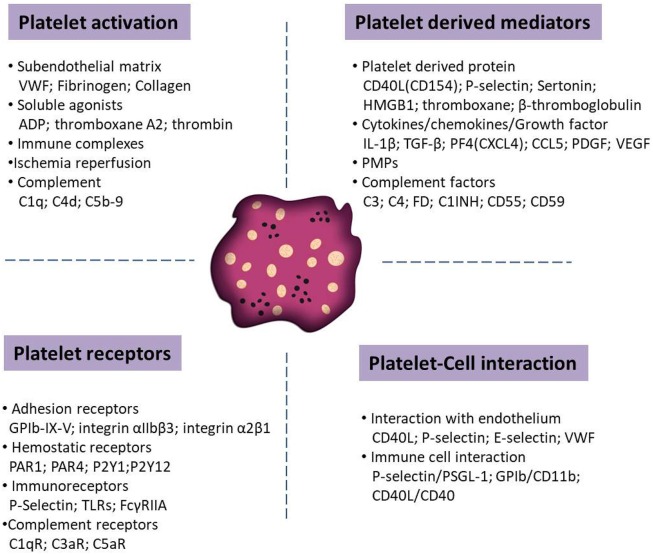
Schematic overview of distinct molecules that tune the function of platelets in autoimmune diseases. Relevant molecules has been categorized into platelet activators, soluble factors released from platelets upon activation, surface receptors that mediate the interaction with other cells and receptors that trigger platelet adhesion and activation.

Upon activation, platelets shed microparticles and they release potent immune modulatory mediators stored in their granules, including proinflammatory cytokines and chemokines (e.g., IL-1ß, TGF-ß, PF4, and PDGF). Platelets are also able to present a number of adhesion (e.g., GPIb-IX-V and P-selectin) and immune receptors (e.g., toll-like or Fc receptors) for prompt responses to the external environment. These receptors enable platelets to interact with activated vascular endothelial cells and immune cells, such as neutrophils, monocytes and lymphocytes. Context dependent, these interactions may tune hemostatic and immune responses, including the activation of the complement system. Figure 1 summarizes various molecules mediating platelet functions in autoimmune diseases.

### Platelet Granules and Platelet Derived Microparticles (PMPs)

There are three types of platelet granules: α-granule, dense granule and lysomal granule. Upon platelet stimulation, granules undergo rapid secretion of their contents into the extracellular space.

The most plentiful (40–80 per platelet) and largest platelet granules (200–400 nm) are α-granules (18). They store almost 300 different proteins, including chemokines, cytokines, growth factors, and adhesion receptors (21–23). However, it is of note that the release of these bioactive substances is not random but dependent on the stimulus (20). Recent observations suggest that platelets contain distinct subpopulations of α-granules which facilitate the differential release of specific α-granule components during platelet activation (24, 25). These secretion products do not only contribute to hemostasis and thrombosis but do also play a potential role as immune mediator by amplifying inflammatory responses (16). Several α-granule derived molecules are frequently reported in the context of skin autoimmune diseases, such as complement factors, CD40L, platelet factor 4 (PF4, also known as CXCL4), and P-selectin (19, 20, 26).

The complement is a complex innate immune system for pathogen defense. The dysregulation of the complement mediates excessive inflammation and tissue injury (27–30). Interestingly, platelet α-granules contain a broad spectrum of complement molecules (31–33). For example, platelets store C3, C4, and factor D which are important components of the complement cascades (31, 34). Also complement attenuating factors C1 inhibitor, CD55, and CD59 are secreted upon platelet activation (35).

CD40L is a transmembrane protein of the TNF superfamily. Under quiescent conditions, CD40L is stored in α-granule whereas it gets exposed on the surface during platelet activations. Upon shedding, CD40L could be released as soluble protein (sCD40L) with cytokine-like activities. CD40L interacts with CD40 on dendritic cells (DCs), B cells and T cells, inducing DC maturation (36), T cell activation (37, 38), B cell isotype switching and antibody production (39). This suggests a significant role of the CD40-CD40L axis in regulating the innate and adaptive immune responses (40). Platelet derived CD40L has also been shown to induce tissue factor expression in monocytes (40), which contributes to the activation of the extrinsic coagulation cascade. Moreover, platelet CD40L can bind to CD40 on endothelial cells, inducing the up-regulation of adhesion molecules (E-selectin, VCAM-1, and ICAM-1), chemokines (IL-8 and CCL2) secretion (22, 41), and VWF release (6). Thus, CD40-CD40L signaling further promotes the adhesion and extravasation of leukocytes at the site of platelet activation. Interestingly, constitutive expression of CD40 at the surface of platelets can further mediate platelet CD62P expression and granule release after perception of CD40L (42).

PF4 is one of the most abundant cytokines in α-granules and it is a potent antiangiogenic chemokine (43). In addition, PF4 induces the release of proinflammatory cytokines from leukocytes and promotes neutrophil chemotaxis (44, 45).

α granules contain also P-selectin a glycosylated transmembrane protein (46) recognizing carbohydrate moieties comparabler to C-type lectins. Similar to CD40L, P-selectin is translocated during platelet activation to the surface membrane or secreted into the plasma as soluble P-selectin (sP-selectin). P-selectin is a key adhesion molecule supporting the close interaction with other immune cells (20, 22, 47). P-selectin ligand-1 (PSGL-1) is the major receptor for P-selectin and it is expressed mainly on neutrophils and monocytes (48, 49). The cross-linking between P-selectin and its corresponding ligand PSGL-1 plays a pivotal role in the formation of platelet-leukocyte aggregates (50, 51) and in the upregulation of tissue factor expression on monocyte (52). P-selectin-PSGL-1 stimulated platelets could further activate neutrophils to form neutrophil extracellular traps (NETs) (53). However, the impact of P-selectin to induce NET formation remains controversial. Results reported by Clark et.al and Maugeri et.al indicate that P-selectin is dispensable for NET generation (54, 55). NETs reversely enhance blood coagulation by direct interaction with VWF (56) and through platelet activation (57, 58). In addition, platelets which are tethered on endothelial cells act as a bridge to promote the adhesion of neutrophils on the blood vessel wall through P-selectin (59).

Dense granules, smaller and less abundant than α-granules, store small non-protein molecules, such as ADP, ATP, serotonin (5-HT), and calcium (2, 16). Platelet dense-granule secretion plays a critical role in the amplification of platelets responses and thrombosis (60). As a platelet agonist, serotonin can modulate autocrine and paracrine platelet aggregation through the interaction of serotonin receptor on platelets (61). In line with this, a variety of immunomodulatory functions of serotonin have been reported, including recruitment of neutrophils to the site of inflammation, stimulation of chemokine secretion by monocyte and T cell proliferation (62, 63).

In parallel, activated platelets release microparticles by shedding of the plasma membrane (64). PMPs have diameters ranging from 0.1 to 1 μm, which are marked by the expression of surface CD41 (64, 65). Although various cellular lineages are able to release membrane microparticles, PMPs make up the main source in human circulation (65). Diverse platelet components are presented in PMPs, including transcription factors, cytokines, growth factors, lipid mediators, nucleic acid, lipid mediators, and mitochondria (25, 65, 66). Due to the size of PMPs, PMPs have been shown to selectively infiltrate tissues and deliver these bioactive factors to recipient cells, triggering inflammation and thrombosis (26, 64, 67, 68). For example, PMPs deliver CD40L to B cells, inducing efficient B cell response and antibody production (26, 69). Similarly to activated platelets, PMPs bear negatively charged PS and potentially TF on their surface, which supports coagulation via the activation of FX and prothrombin (70, 71). In addition, PMPs have the ability to directly attach to fibrin and enhance the local production of thrombin which further amplifies the thrombus formation (70, 72).

### Platelet Receptors

Platelet has a variety of surface receptors, and the majority of these receptors trigger either platelet activation or platelet adhesion. For example, Platelet glycoprotein complex GPIb-IX-V enables platelet binding to subendothelial and luminal exposed VWF even under high shear stress (73, 74). This interaction is further enforced by collagen or fibrinogen through the platelet receptors GPVI, and integrin α_2_β_1_ or integrin αIIbβ3, respectively (2, 73).

Classic hemostatic agonists (thrombin and ADP) not only mediate hemostasis, but are also directly linked to inflammatory receptor mediated signaling pathways. The G_aq_-coupled protease-activated receptors PAR1 and PAR4 are the two main thrombin receptors on platelets (75). Signaling through these receptors stimulates the Rho-associated protein kinase and phospholipase Cβ, leading to further downstream protein kinase C (PKC) activation and Ca^2+^ release (2, 76). ADP is another potent platelet activator and can be secreted from dense granules upon platelet activation. On the membrane of platelets, the two purinergic receptors P2Y_1_ and P2Y_12_ are expressed, coupling to Gqα and Giα, respectively (77). Signaling via P2Y_1_ mediates PKC activation, Ca^2+^ release into the cytoplasm and induces platelet shape change (78). P2Y_12_ stimulates phosphoinositide3-kinases activation which triggers platelets granule secretion and aggregation (2, 78). Interestingly, PAR signaling promotes also the release of ADP (79, 80), linking these two pathways and enabling autocrine platelet activation. Clopidogrel, a common used drug to prevent heart disease and stroke, blocks the P2Y_12_ on platelets explaining its high efficacy.

Platelets could also directly recognize immunoglobulins and immune complexes (IC) through the Fc receptor FcγRIIA (81, 82). IC binding to FcγRIIA induces platelet hypersensitivity to thrombin stimuli (83). In addition, FcγRIIA activation can also support platelets serotonin release (84).

Toll-like receptors (TLRs) are another group of immunoreceptors expressed on platelets, which enable platelets to recognize endogenous damage associated molecular patterns (DAMPs) and pathogen associated molecular patterns (85, 86). TLR4 is the most abundantly expressed TLR on platelets and it can detect ligands such as lipopolysaccharide (LPS) and high mobility group protein B1(HMGB1) (87). In this context, Clark et al. reported that LPS induces platelet binding to adherent neutrophils, resulting in neutrophil activation and the formation of NETs (54).

Finally, platelets express several complement receptors (CR) (31). Among them, receptors C3aR and C5aR recognize the strong proinflammatory complement effectors C3a and C5a (88). These two receptors have low expression levels on resting platelets but their expression is increased upon inflammatory stimulation (88, 89). Notably, P-selectin contains nine consensus domains which are common to the structural motif of CRs (90). Therefore, apart from its function as adhesion molecule, P-selectin may also mediate complement effector binding to the surface of platelets to support complement activation (90). Moreover, the surface expression of C1q receptors on platelets has been linked to the initiation of the classical complement pathway activation (91–93).

## Platelets Role in Skin Autoimmune Diseases

### Systemic Lupus Erythematosus (SLE)

**Figure 2:**
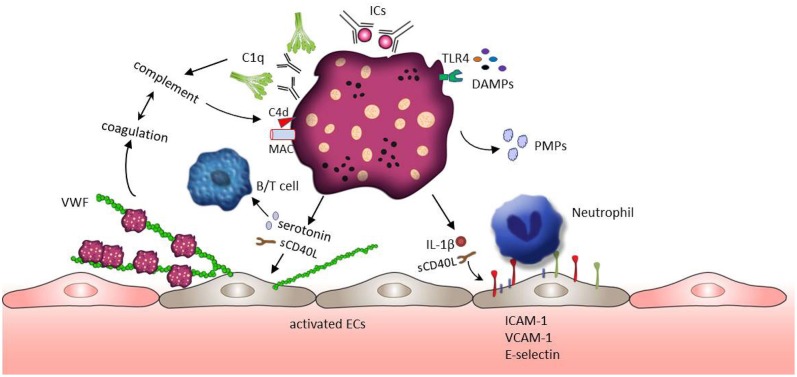
Platelet activation and subsequent effects in SLE. ICs and DAMPs (e.g., HMGB1 or S100A8/9), both enriched in the blood of SLE patients, activate platelets through binding to platelet surface receptors FcγRIIA and TLRs. ICs further induce the complement system activation leading to the deposition of complement fragments (e.g., C1q,C4d, MAC) on the platelet surface which in turn further potentiates platelet activation. Activated platelets release their contents such as IL-1β, sCD40L, and serotonin. These factors mediate the up-regulation of adhesion molecules (e.g., E-selectin, VCAM-1, and ICAM-1) on endothelial cells, promoting the adhesion of immune cells. In addition, sCD40L and serotonin can stimulate the release of VWF from endothelial cells, but also support B cell and T cell activation. VWF mediated trapping and activation of platelets initiate coagulation which may further foster the activity of the complement system building a positive feedback loop.

Systemic lupus erythematosus is a chronic autoimmune disease characterized by systemic inflammation in many different organs. SLE is also associated with thrombotic complications and increased cardiovascular morbidity (94, 95). A wide range of research on the pathogenesis of SLE focus on the formation of autoantibodies and autoantibody induced IC, as well as the dysregulation of lymphocyte function and activation of the complement system (95). However, platelets also play an important role in inflammatory activity and immune response. Growing evidence indicates that platelets are activated in SLE patients and contribute to the pathogenesis of SLE (25, 26). Moreover, thrombocytopenia is a common hematologic manifestation in SLE and associated with severe SLE abnormalities such as neurological abnormality and kidney injury (96, 97). As a promoter of complement activation, the presence of antiphospholipid antibodies (aPLs) is often detected in the patients with thrombocytopenia. The mean platelet volume (MPV) is widely used for assessing platelet activation in various inflammatory conditions. However, the current literature on MPV in SLE is contradictory and the usefulness of MPV as a biomarker in SLE still need to be explored (20, 98–101). On the molecular level, the production of thromboxane, P-selection expression and the release of sCD40L, PMPs and β-thromboglobulin have been reported as markers for platelet activation. Their levels are increased in SLE patients and associated with an increased risk of thrombotic events (102–108). The role of platelets in the SLE pathophysiology is depicted in Figure 2.

Several pro-inflammatory mediators are responsible for platelet activation in SLE patients. Among them, DAMPs such as HMGB1, or S100A8/9, both enriched in the blood of SLE patients (109–111), might have the potential to trigger platelet activation through TLR4 signaling. Platelet activation is also mediated by the recognition of SLE-associated ICs through FcγRIIA receptors (25). In addition, TLRs (TLR7, TLR9) exposed at the platelet surface bind to RNA-containing ICs or nucleic acid (ssRNA, dsDNA), contributing to platelets activation (112). ICs are frequently formed by aPLs on the surface of platelets (in almost 40% of the SLE patients) (113, 114). Those platelet-bound ICs could be detected by the complement system mediating the deposition of C4d and the formation of the membrane attack complex (MAC) on the platelets' surface (113, 115). As mentioned above, platelets actively contribute to the complement cascade through the production of several complement factors including C1q, C3, and C4. These complement effectors in turn potentiate the impact of platelets on inflammation (32). For example, MAC promotes platelets to release proinflammatory mediators such as serotonin, thromboxane and β-thromboglobulin stored in their granules (116, 117). Fixation of C4d on the platelet surface supports platelet aggregation and platelet interactions with monocytes and endothelial cells in the context of venous thrombosis (31, 32, 113, 115). Platelets marked with C4d are detected in almost 20% of patients with SLE suggesting C4d positive platelets as a prognostic biomarker (113, 115, 118). The deeply interwoven connection between the complement system and the coagulation (119, 120) has been emphasized by the work of Kölm et al. (121). In their recent study it was demonstrated that VWF bind to C1q and that the C1q-VWF complexes induce platelet adhesion in correlation with the frequently observed thrombotic events in SLE patients (121).

Next to the involvement of the complement system, ICs were shown to mediate the release of serotonin (84, 122, 123), PMPs (108, 124), and IL-1β (106) from platelets. Released serotonin disturbs the endothelial barrier in SLE promoting an increase in vascular permeability (25, 125). Serotonin also has a major effect on T cell activation and proliferation through the interaction with the lymphocyte 5-HT7 receptor (63). Beside serotonin, SLE patients display higher concentrations of PMPs. A study involving 60 SLE patients shows that platelets are the main source of circulating microparticles in SLE (124). Interestingly, those PMPs are C1q+ and can form IC with IgG and IgM (124). Levels of PMPs IgM conjunctions are negatively correlated with SLE severity (124). In contrast, levels of IgG+ PMPs are positively associated with SLE activity (124). IgM autoantibodies bind to apoptotic cells in patients with inactive SLE and may facilitate non-inflammatory removal of PMPs by monocytes or macrophages (124, 126). However, the PMPs-IgG+ stimulate monocytes promoting the expression and release of pro-inflammatory cytokines such as IL-1β, TNF-α, and IFN-α (124). Additionally, IL-1β can also be released from activated platelets. IL-1β induces the expression of NFκB driven inflammatory genes, such as, IL-6 IL-8 and ICAM-1 in endothelial cells which in turn mediates immune cells recruitment and immune-thrombotic complications (106, 127). Endothelial activation could also be directly triggered through platelet derived CD40L (6, 41). This acute activation is characterized by Weibel-Palade body exocytosis, release of VWF multimers and thus the rapid recruitment of further platelets which promotes the sequestration of circulating monocytes by the P-selectin-PSGL-1 interaction (128).

Activated platelets also contribute to the regulation of adaptive immune responses. Platelets are the major source of sCD40L in the circulation (25, 105) and the CD40L signaling through its receptor CD40 on B cells and T cells lead to immunoglobulin IgG and IgM synthesis and the germinal center reaction (38, 39). As reported by several groups, the CD40/CD40L axis promotes DC maturation and IFNα secretion (36). IFNα is a key cytokine in the pathogenesis of SLE indicated by increased levels of the IFNα regulated proteins PRKRA, IFITM1 and CD69 in platelets from SLE patients. The up-regulation of the IFNα system is strongly associated with vascular disease in SLE (20, 129).

### Systemic Sclerosis (SSc)

**Figure 3:**
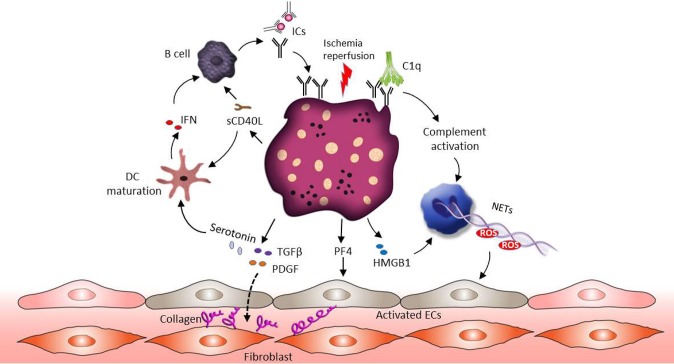
The role of platelets in SSc pathophysiology. Ischemia-reperfusion injury associated endothelial cell damage induces platelet activation. Similar to SLE, the presence of ICs could promote hypersensitivity of platelets and complement activation. Activated platelets release their profibrotic mediators such as TGF-β, serotonin and PDGF, and these factors stimulate connective tissue fibroblasts proliferation and increase collagen production. In addition, both HMGB1 released from platelets and complement activation can support NET formation and ROS release, which further mediates vascular endothelial dysfunction. Apart from NETs, platelets derived PF4 activates endothelial cells and induces SSc associated vascular damage. Activated platelets also release sCD40L to active B cells through CD40 signaling, which leads to B cell auto-antibody production. Moreover, sCD40L and serotonin can promote the maturation of myeloid DCs, followed by IFN production and further B cell activation.

SSc is an autoimmune disease characterized by excessive connective tissue deposition and fibrosis, vasculopathy and a dysregulated immune system (130, 131). Enhanced platelet activation and aggregation can be observed in patients suffering from SSc (131–133). Ischemia reperfusion alternation associated with Raynaud's phenomenon in fingers is frequently the first manifestation of SSc (132). Ischemia-reperfusion injury promotes endothelial cell damage, which results in the release of reactive oxygen species (ROS), DAMPs, the activation of the complement and the exposure of collagen to the blood flow (134–137). This inflammatory environment stimulates platelet recruitment and activation. Similar to SLE, the presence of aPLs related ICs could further promote hypersensitivity of platelets (138). In this context, a specific increase in the number of platelet non-integrin type I collagen receptor was detected in SSc patient's platelets (139). This may explain the accelerated aggregation status of SSc patients' platelets when exposed to collagen. The pathophysiological role of platelet in SSc is shown in Figure 3.

Extensive experimental evidence supports the notion that platelets participate in the fibrotic process mainly by releasing profibrotic mediators (131, 140). Transforming growth factor β (TGF-β) and platelet-derived growth factors (PDGFs) are thought to be the central pathogenic profibrotic mediators. Activation of the TGF-β pro-fibrotic signaling pathway has long been implicated in fibrotic diseases, including SSc (141, 142). Circulating platelets contain high concentrations (about 40–100 times higher compared to other cells) of TGF-β in their α-granules and release it rapidly upon activation (143–145). Several studies showed that TGFβ plays a pivotal role in extracellular matrix remodeling through the control of the collagen synthesis, as well as secretion of fibronectin and thrombospondin-1 (141, 146). Notably, TGF-β stimulates the production of a plethora of secondary mediators from fibroblasts, such as connective tissue growth factor and endothelin-1, modulating the downstream fibrotic signaling activation (141, 147, 148). Similar to TGF-β, activated platelets can also release PDGF, which is able to stimulate connective tissue fibroblasts proliferation and increase collagen production (131, 149). A study revealed that PDGF-A is increased in the dermal interstitial blister fluid of SSc patients (150) and blockade of PDGF receptors by Crenolanib is effective in reducing skin fibrosis in preclinical models of SSc (151). Additionally, Nintedanib, a tyrosine kinase inhibitor, inhibits the PDGF and TGF-β induced activation of SSc fibroblasts and prevents the onset of the disease in different mouse models (152, 153). As mentioned above, platelets are the main source of serotonin in the circulation and the profibrotic role of serotonin has received significant attention recently (154). Serotonin could enhance collagen production in fibroblasts through the 5HT-2B receptor, which is over expressed in SSc patients' skin (154). Platelets in SSc further contribute to skin fibrosis because they interact with dermal microvascular endothelial cell which induces the subsequent secretion of profibrotic mediators such as thymic stromal lymphopoietin (155).

The involvement of the endothelium in patients suffering from SSc is mirrored by an increased risk of vascular disease such as Raynaud's phenomenon, pulmonary arterial hypertension, ischemic ulcer due to vascular damage, renal and cardiac disease (131, 156). Several mechanisms have been suggested to explain the role of platelets in SSc associated vasculopathy. Experimental data have shown that platelets derived PF4 could enhance the expression of thrombospondin-1 and promote endothelin-1 secretion from human endothelial cells, resulting in an inflammatory phenotype of endothelial cells and SSc associated vascular damage (157). Similarly to SLE, the existence of PMP is abundant in the blood of SSC patients, especially HMGB1-associated PMPs (158–160). HMGB1 released from activated platelets in SSc patients, sustains autophagy associated activation of neutrophils in SSc and commits them to generate NETs, leading to vascular endothelium dysfunction (158). Recent studies suggest that antiangiogenic factors such as VEGF165b, together with proinflammatory (CD40L) and profibrotic (TGF-β) factors secreted by platelets, can contribute to the progression of peripheral microvascular damage and defective vascular repair in SSc (161).

It is becoming increasingly clear that platelets act as key regulators of the immune response participating in the pathogenesis of SSc. A large array of proinflammatory mediators are either synthesized within platelets or stored in the granules and released upon activation. Platelets derived CCL5, PF4, CXCL5, and leukotriene-B4 hold important leukocyte chemoattractant properties, recruiting neutrophils, monocytes, and fibroblasts to the site of inflammation, which in turn amplify local inflammatory reactions (132, 162). What is more, it has been shown that PF4-activated monocytes trigger ROS production and the release of the procoagulant VWF in endothelial cells (163). Also P-selectin translocates to the platelet membrane and forms an adhesive bridge between endothelial cells and neutrophils, monocytes and T cells, thereby facilitating the formation of heterotypic platelet-leukocyte aggregates on the surface of blood vessel (162). Activated platelets express and release sCD40L which accounts for almost 90% of circulating sCD40L (164). The interaction between sCD40L and CD40 on B cells leads to increased immumoglobulins production and B cell proliferation, highlighting the remarkable role of platelets in the regulation of adaptive immune response (39). Another important SSc related immune mediator is serotonin (63). Apart from the role in the regulation of collagen production, serotonin also enhances T cell activation and proliferation through 5-HT7 receptor signaling (165). Some studies also suggest the immunomodulatory properties of serotonin through the maturation of myeloid DCs (166) and the regulation of the production of proinflammatory mediators such as IL-6 and TNF-α from monocyte (167).

### Antineutrophil Cytoplasmic Antibody (ANCA)—Associated Small Vessel Vasculitis

**Figure 4:**
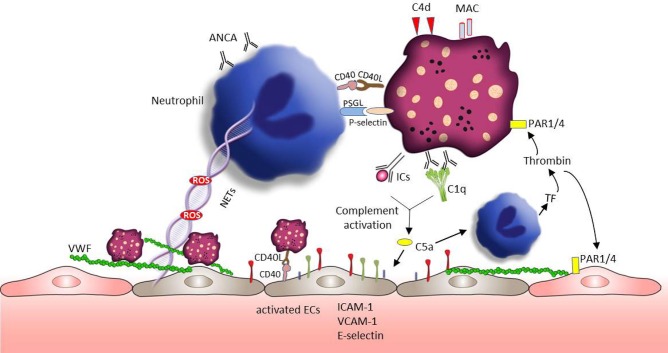
Platelets involvement in the pathogenesis of AAVs. Neutrophils play a crucial role in the development of AAVs. Platelet receptors CD40L and P-selectin enable the interaction with neutrophils promoting ROS generation and NET formation. ROS and NETs are toxic to the endothelium and can lead to VWF release and vascular injury. VWF is well-known to mediate platelets adhesion and aggregation. Similar to neutrophils, sCD40L-CD40 can also mediate platelet -endothelial cell interaction, which can enhance the expression of endothelial cell adhesion proteins (e.g., E-selectin, VCAM-1, and ICAM-1). C5a, a chemoattractant anaphylatoxin, can directly attract and activate neutrophils promoting the exposure of tissue factor. Tissue factor initiates the plasmatic coagulation which culminates in the generation of thrombin. Thrombin is able to further activate platelets and endothelial cells through PAR signaling pathway, followed by platelet degranulation and VWF release. In addition, C5a can also directly active endothelial cells accelerating vascular injury.

Vasculitis represents a group of complex diseases with the pathology of blood vessel wall inflammation (168, 169). Based on the size of vessels involved, vasculitis is categorized by large, medium, and small vessel vasculitis (170). The pathogenesis of vasculitis remains incompletely understood and few studies reported about the role of platelet in large vessel vasculitis. Our review will focus on ANCA-associated small vessel vasculitis (AAVs), which are characterized by the inflammatory cell infiltration of small sized vessel walls in multiple organ systems (171). AAVs also display a broad variety of cutaneous manifestations, including tissue necrosis, and vascular destruction (172). The neutrophil derived proteins myeloperoxidase and proteinase 3 are the main target antigens of ANCA (173). Platelets are first responders during vascular injury and they are inflammatory effector cells closely correlated with the activity of vasculitis (7, 174). In line with that PMPs containing proinflammatory cytokines are increased in AAVs patients and supposed to trigger an acute inflammatory state in AAVs (175). Figure 4 summarizes the platelet activation and subsequent effects in AAVs.

CD40 and CD40L are expressed on endothelial cells, platelets and epithelial cells and their signaling mediates several processes of vascular inflammation. sCD40L-CD40 mediated platelet-endothelial cell interaction, induces cytokine and growth factors production (such as IL-1β, TNF-α, IL-2, VEGF) in endothelial cells and enhance the expression of endothelial cell adhesion proteins (such as ICAM-1, VCAM-1, P-selectin) (40, 41). This further leads to the recruitment of neutrophils and lymphocytes to the site of injury. sCD40L also enhances endothelial expression of tissue factor (176) and contributes to the endothelial dysfunction through ROS generation (177, 178). Neutrophils are strong mediators of the pathogenesis of AAVs. sCD40L can also directly activate neutrophils, mediating ROS release (179) and macrophage 1 antigen (CD11b/CD18) expression (180). Notably, activated platelets can stimulate NET formation by the release of sP-selectin (53). In turn, NET fibers could bind platelets supporting their aggregation (181). Additionally, NET components, such as histones, can further stimulate platelet and endothelial cell activation (181–183). Blocking of neutrophil PSGL-1 completely inhibits the activated platelet mediated NET formation (53). Similar to SLE and SSc, the development of vasculitis is at least partially related to the activity of the complement system (184). The activation of the complement alternative pathway both in the fluid phase and on the surface of platelets leads to MAC deposition on platelets and generation of the inflammatory factors C3a and C5a (185). Both complement effector molecules elicit the expression of cellular adhesion molecules such E-selectin, ICAM-1, and VCAM-1 on endothelial cells and the production of cytokines/chemokines and related receptors (such as VEGFC-R, IL-6R, IL-18R) (186). Because of the presence of C3aR and C5aR on platelets surface, C3a and C5a can also promote the activation of platelets, supporting coagulation and inflammation in AAVs (32, 185). C5a may also trigger the release of tissue factor expressing microparticles and ROS from ANCA sensitized neutrophils (187). Tissue factor, as a pivotal part of the coagulation cascade, catalyzes the generation of thrombin, indicating again the close connection between the complement and the coagulation system. Thrombin activates platelets promoting platelet degranulation and the release of P-selectin. In addition, thrombin can also induce the release of VWF from the endothelium mediating platelet adhesion and aggregation amplifying the cross-talk between coagulation and the innate immune system.

### Other Skin Autoimmune Diseases

A limited number of studies have reported on the potential involvement of platelets in other skin autoimmune diseases such as chronic urticarial (CU), vitiligo, bullous pemphigoid (BP), and psoriasis.

In CU patients the assessment of MPV as a supposed marker for platelet activation provided conflicting results (188–190). Contradiction might have been due to the variations in patients' selection, the stage of the disease and patients' disorders (such as obesity and diabetes) during the laboratory analysis (20, 191).

Some studies investigated the intradermal injection of platelet rich plasma (PRP) into vitiligo patients (192). PRP is an autologous blood-derived product with enriched platelets and high concentration of growth factors secreted from platelet α –granules (193, 194). These factors can stimulate melanocyte migration and promote keratinocytes proliferation. Several research groups reported the combination of PRP treatment with narrowband–ultraviolet B phototherapy or exposure to fractional CO_2_ laser light results in a significant improvement in repigmentation, improving patient compliance (194–196). However, the limitations of those studies are the small sample size and short follow-up periods.

BP is a common autoimmune bullous disease, characterized by autoantibodies directed against hemidesmosomal proteins (BP180 and BP320) of the skin followed by subepidermal blistering (197). The pathogenesis of BP is not fully understood, and the majority of research that has previously address the pathogenesis of BP focused on the immune response caused by autoantibodies. Research about the role of platelet in BP is scarce. A study found significantly higher number of eosinophil and MPV values in BP patients, pointing to a disease related platelet activation (198). Another study discovered increased levels of sP-selectin in BP patients suggesting also a potential platelet activation (199). However, further studies are required to better understand the contribution of platelets on BP.

Psoriasis is a chronic autoimmune-mediated skin disease characterized by red scaly plaques. Some previous studies showed a close association between platelets activation and psoriasis activity. *In vitro*, platelets from psoriasis patients are more sensitive to thrombin and ADP (200, 201). In line with this increased sensitivity, plasma levels of platelet activation markers such as ß-thromboglobulin, PF4, PMPs and sP-selectin were also elevated in psoriasis patients (202, 203). Another recent study evaluated 320 patients with psoriasis vulgaris and found that the mass of platelets is increased in the affected patients (204). Interestingly, platelet activation was also attributed to the increased endogenous antimicrobial cathelicidin LL37 in psoriasis patients (205). In platelet, LL37 can induce enhanced fibrinogen binding, p-selectin exposure and Ca^2+^ mobilization (206). In a murine psoriasis model, the P-selectin/PSGL-1 interaction was shown to promote the formation of platelet-leukocyte aggregates and to favor leukocyte rolling in murine skin microvasculature (207). Similarly, Teague et al. showed a disease severity dependent interaction between low density of neutrophils and platelets in psoriasis patients (208).

### Platelets as Potential Biomarker for Monitoring and Diagnosis of Skin Autoimmune Diseases

Platelet derived components and platelet indices can potentially be used to monitor and diagnose autoimmune diseases. In SLE, the serum or plasma levels of platelet associated molecules, such as HMGB1, S100A8/A9, sCD40L, and CCL5 as well as platelet derived PMPs harboring IgGs have been shown to correlate with the SLE disease activity index (SLEDAI) score (105, 110, 124, 209, 210). Lood et al. have recently correlated platelet derived S100A8/A9 with cardiovascular complications in SLE patients (109). Apart from platelet released molecules also the presentation of P-selectin at the surface of platelets has been associated with the severity of SLE (106). Next to an active P-selectin exposure, also the deposition of C4d on the platelet surface has been correlated to the occurrence of aPL-related venous thrombosis and to the SLEDAI score (115, 211). Notably, in a cross-sectional study of 105 patients with SLE, the authors reported that compared with healthy individuals, platelet C4d was 100% specific for SLE patients (115). As further demonstrated in a retrospective cohort study, platelet C4d is significantly associated with all-cause mortality and ischemic stroke in SLE patients (211). Therefore, C4d bound platelet can be used as a reliably biomarker for SLE and a predictor for thrombotic events in SLE patients.

In SSc, the levels of platelet released mediator such as serotonin, sCD40L, sP-selectin, HMGB1, and PDGF have been connected to the severity of the SSc related fibrosis or vasculopathy (151, 154, 212–214). Moreover, increased numbers of PMPs have also been observed in SSc patients (158). These few reports suggest the potential usage of platelets and platelet associated compounds as prognostic biomarker, however further validating research is required.

Tomasson et al. reported that sP-selectin and sCD40L released by platelets are positively associated with vasculitic activities (215). Platelet counts are increased in the active stage of AAVs, which can be used to distinguish active disease from acute systemic infections (176). In line with SLE, SSC, and AAV, elevated sP-selectin levels were also previously detected by several groups in patients with CU, especially in autologous plasma skin test positive patients and those which are aspirin-intolerant (216). In addition, the sP-selectin levels correlated positively with the urticarial severity score (217), indicating platelet activation as a possible indicator of the CU disease activity. In psoriasis, several studies reported that the plasma PMPs and P-selectin levels were significantly correlated with the psoriasis area and the severity index (PASI) score (203, 218). Recently, Raghavan et al. proposed a strong correlation between the PASI score and MPV values and platelet counts (219). However, further research is required to confirm the usability of platelet related parameters for the diagnosis and the monitoring of AAV, CU, and psoriasis.

### Platelets as Therapeutic Targets in Skin Autoimmune Diseases and Future Perspectives

**Figure 5:**
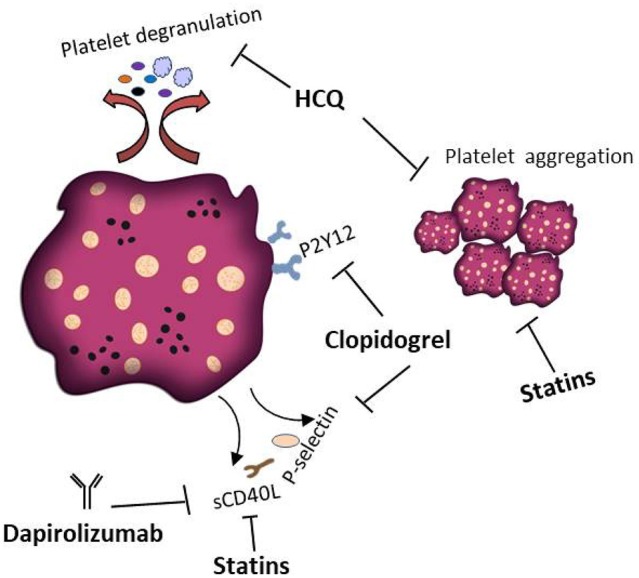
Platelets as therapeutic targets in skin autoimmune disease. Several treatments that interfere with the pathophysiological activity of platelets in skin autoimmune diseases have been proposed. HCQ inhibits platelet aggregation and degranulation. Clopidogrel is a P2Y12 receptor inhibitor preventing the release of platelet derived sCD40L and P-selectin. Dapirolizumab is a monoclonal antibody specifically blocking sCD40L functions. Statins are supposed to affect the thrombotic activities of platelets and to decrease the plasma levels of sCD40L.

A number of small molecule inhibitors and monoclonal antibodies have been developed to target platelet activation in skin autoimmune diseases. For example, hydroxychloroquine (HCQ) is an antimalarial compound that provides an effective treatment (220) in all types of Lupus erythematodes. One beneficial mechanism of HCQ therapy is the inhibition of platelet aggregation and degranulation (221, 222). HCQ mediated platelet inhibition is also effective for the treatment of SSc (223). Another therapeutic option to block platelet activation is the use of the P2Y12 receptor inhibitor clopidogrel (224). Several studies reported that in lupus-prone mice clopidogrel treatment inhibits the release of platelet derived sCD40L and P-selectin (36, 225). In SSc mice models, clopidogrel has been shown to decrease fibrosis (154). However, one study reported that a standard dose of clopidogrel (75 mg) inhibits platelet activation in SSc patients but had no effect on plasma serotonin levels. Clopidogrel treatment even led to a significant increase in soluble VCAM-1, indicating endothelial dysfunction as adverse effect (226). Additionally, fibrosis has been successfully managed in an SSc murine model using the serotonin receptor inhibitors terguride, cyproheptadine, and SB 204741 (154, 227). Dapirolizumab is an anti-CD40L Fab antibody fragment and currently evaluated in a phase 2 clinical trial (228). The treatment exhibit an excellent tolerance profile and convincing clinical efficacy in SLE patients. Notably, no thromboembolic adverse events occurred in the phase 1 clinical trial (228). CD40L signaling is also inhibited by Statins, because this class of small molecular compounds is able to reduce the CD40L content in platelets and thus sCD40L levels (229, 230). Therefore, treatment of SLE patients with statins can decrease platelets thrombotic activities (231). Platelet inhibition as therapy of AAV has to our knowledge never been investigated. Figure 5 summarizes current treatment options targeting platelets in skin autoimmune diseases.

As future perspectives, several potential treatment options targeting platelet components or preventing platelet activation might be worth to be investigated. For instance, because P-selectin plays an important role in the pathogenesis of autoimmune diseases, Inclacumab, a novel monoclonal antibody against P-selectin (232), might be considered for the treatment of skin autoimmune diseases in future. As discussed in the previous section, C4d deposition on platelets was found to be highly specific for the diagnosis of SLE, and C4d positive platelets are regarded as a risk factor for thrombotic event in SLE patients (115). In line with this, inhibition of the complement system by eculizumab, a C5 neutralizing monoclonal antibody, prevented thrombotic microangiopathy in SLE (233). Therefore, it would be highly interesting to further investigate the treatment effects of C4 neutralizing antibodies or to develop specific compounds preventing the deposition of C4d on the surface of platelets.

Platelets activation is controlled by a plethora of distinct pathways and a better understanding of these signaling routes may open new therapeutic perspectives. Such as, collagen triggers the phosphorylation of Bruton's Tyrosine Kinase (BTK), and signal transducer and activator of transcription 3 (STAT3) can modulate platelets aggregation and activation (234, 235). BTK inhibitor RN486 has been reported to abrogate the generation of PMPs (234) and the STAT3 inhibitor SC99 was effective to inhibit thrombin-induced P-selectin expression and platelet activation (235). In addition, activated platelets are the main source of sCD40L and blockade of the CD40–CD40L interaction by monoclonal antibodies appears to be a promising treatment of autoimmune diseases. Apart from anti-CD40L antibodies, a CD40-targeting peptide was designed recently (236). This peptide mimics the CD40L domain critical for the interaction with CD40 and it has been shown to effectively block CD40-CD40L signaling (236). In a comparable approach, Chen et al. identified small molecule compounds that can interfere with the CD40–CD40L protein-protein interaction (237). To which extend those drugs are applicable to treat autoimmune diseases remains elusive. However, the development of platelet inhibiting compounds is an emerging field of research and it envisions the discovery of novel compounds efficient for the treatment of skin autoimmune diseases.

## Conclusions

Platelets are the main participants of hemostasis and have multiple immunoregulating functions linking coagulation and inflammation. It is becoming apparent that platelet activation might be a biomarker of skin autoimmune diseases activity. Multiple compounds in circulation, such as ICs, DAMPs, VWF and collagen, can stimulate continuous platelet activation. Platelet activation appeared to be further potentiated by the deposition of complement factors at their surface. However, more details about the crosstalk between platelets and complement still needs to be investigated. Upon activation, platelets release a variety of pro-inflammatory mediators such as sCD40L, P-selectin, serotonin, PDGFs, and TGF-β. These released factors contribute to the immune response, ultimately resulting in chronic inflammation and local tissue damage. Activated platelets modulate the function of the innate and adaptive immune system by secreting immune mediators or through direct cellular interactions with immune cells, such as DC, neutrophils, B and T cells. However, the interplay between platelets with other immune cells such as mast cells and monocytes requires further clarification. It would be also worth to investigate novel platelet derived biomarkers to predict and assess the status of the respective disease. There are still some controversies on the assessment of the commonly measured MPV in autoimmune diseases. Contradictive results suggest that only MPV measurements are not sufficient to evaluate platelet function. Platelet activation is a variable and dynamic process and research addressing the role of platelets in autoimmune disease should be not limited to distinct platelet activation processes or distinct factors released by platelets. Right now, targeting platelets using anti-CD40L monoclonal antibodies and ADP receptor inhibitors has been reported to reduce autoimmune reactions in several clinical trials addressing autoimmune diseases. A better understanding of the role of platelets in autoimmune diseases will reveal new therapeutic options in the future.

## Author Contributions

XL, CG, and SS contributed to reviewing the current literature and writing of the manuscript.

### Conflict of Interest Statement

The authors declare that the research was conducted in the absence of any commercial or financial relationships that could be construed as a potential conflict of interest.
